# Supplementary material for: N6-methyladenosine modification is not a general trait of viral RNA genomes
Source: Nat Commun. 2024 Mar 11;15:1964. doi: 10.1038/s41467-024-46278-9 (PMC10928186; doi:10.1038/s41467-024-46278-9)
Supplement: Supplementary file 1 — Supplementary Information [file 41467_2024_46278_MOESM1_ESM.pdf]

N[6]-methyladenosine modification is not a general trait of viral RNA genomes

Baquero-Pérez *et al*.

Supplementary Information

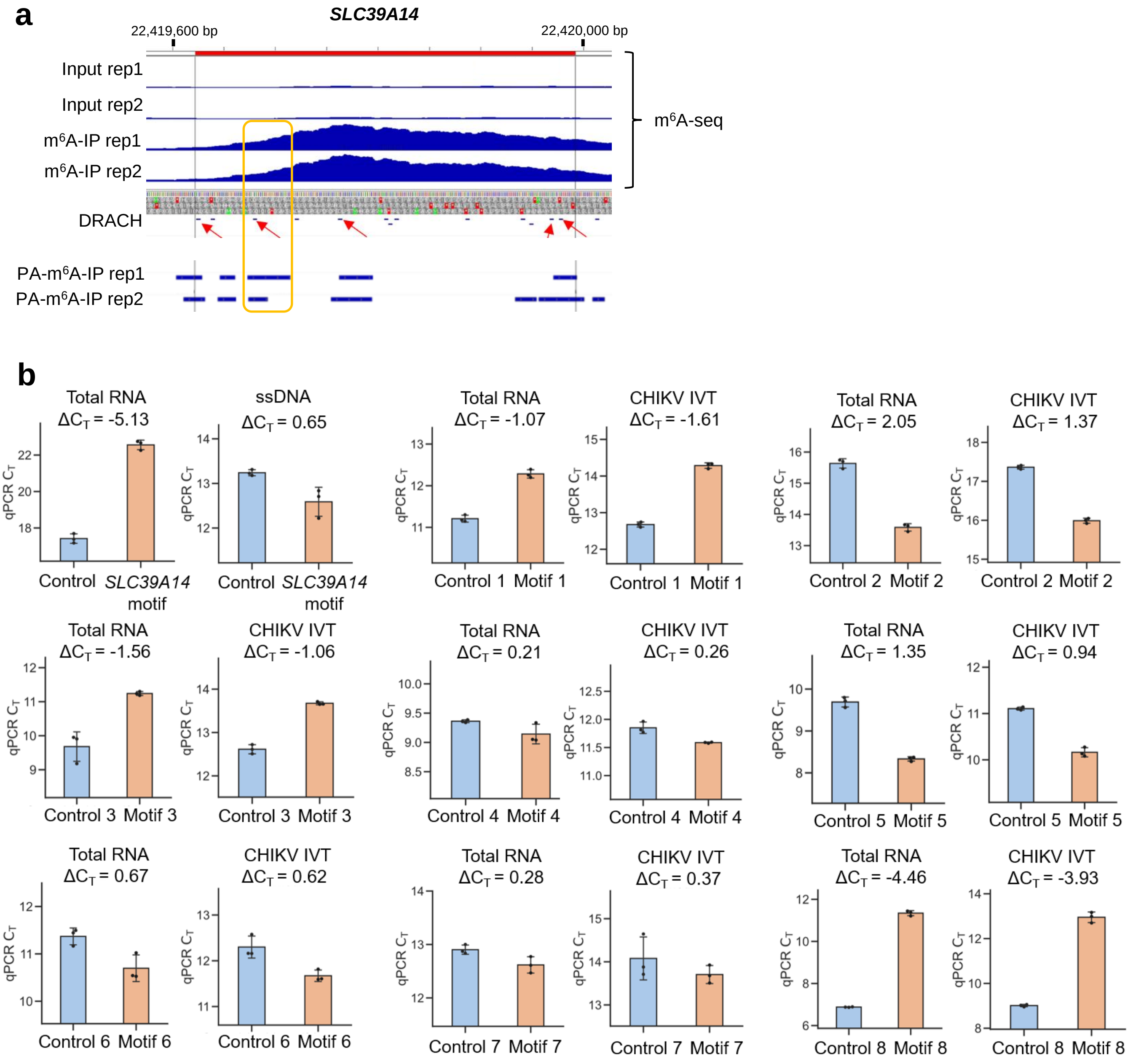

Supplementary Fig. 1. Raw C_T cycles from SELECT analyses of CHIKV RNAs. a) Genome browser tracks display our m6A-seq dataset along with publically available PA-m6A-seq datasets[27]. Mapped reads for the *SLC39A14* transcript are shown. DRACH motifs are depicted as blue thin lines spanning the transcript. Red arrows indicate regions where DRACH motifs overlap with both biological replicates of PA-m6A-seq. The positive control modified adenosine in the *SLC39A14* mRNA (genomic coordinate Chr8:22419678) is highlighted with a yellow line. **b)** The bar chart presents mean values obtained from three independent SELECT reactions, with the error bars representing the standard deviation (SD.). Results are representative from two independent infections.

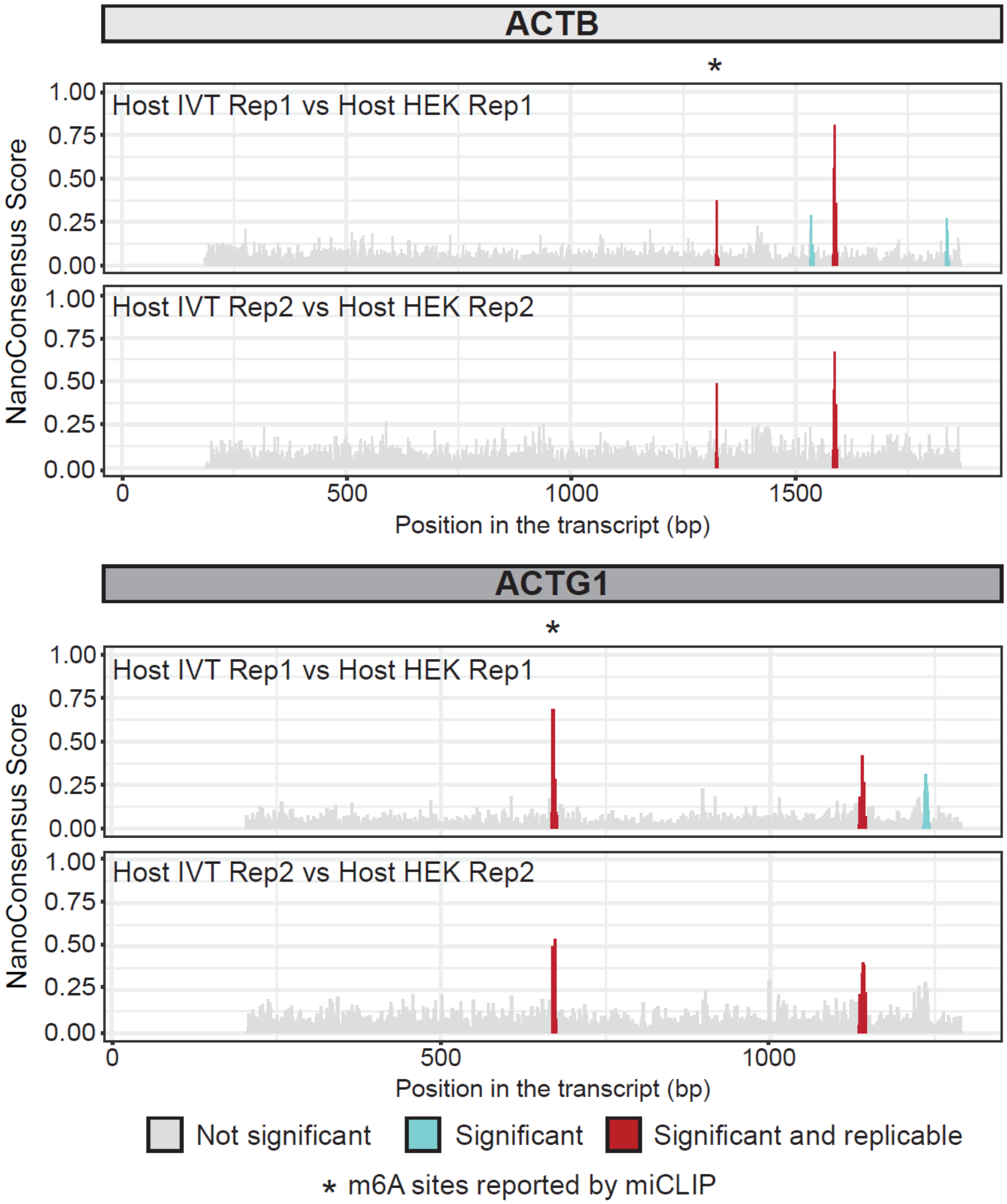

Supplementary Fig. 2. DRS identifies modified sites in host mRNAs. *NanoConsensus*' scores across the human transcripts *ACTB* (upper panels) and *ACTG1* (lower panels) when comparing reads from the CHIKV-infected samples and an IVT human transcriptome. *NanoConsensus´* default parameters were used. In grey, non-significant positions; in blue, regions identified by *NanoConsensus* in only one replicate; in red, regions identified in both replicates. m^6A sites previously identified by miCLIP[28] are indicated with an asterisk (*).

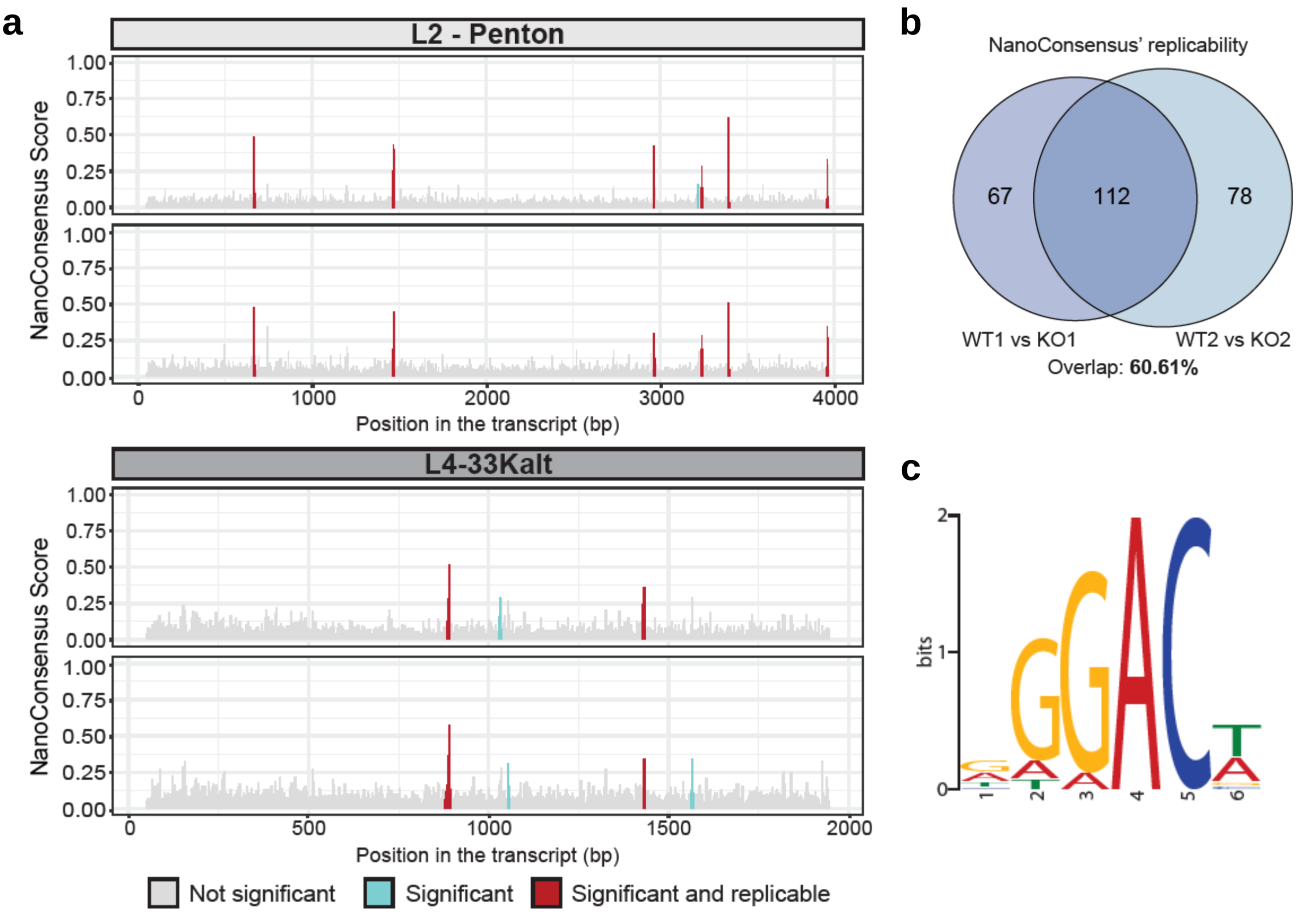

Supplementary Fig. 3. *NanoConsensus* can identify modified sites in the Ad5 transcriptome. a) *NanoConsensus*' scores across adenovirus' transcripts L2-Penton (upper panels) and L4-33Kalt (lower panels) when comparing reads from wild-type and METTL3 knockout samples. *NanoConsensus´* default parameters were used. In grey, non-significant positions; in blue, regions identified by *NanoConsensus* in only one replicate; in red, regions identified in both replicates. **b)** Venn diagram showing the replicability of sites predicted by *NanoConsensus* across comparisons. **c)** Motif significantly enriched in replicable sites identified by *NanoConsensus* in the adenovirus' transcriptome.

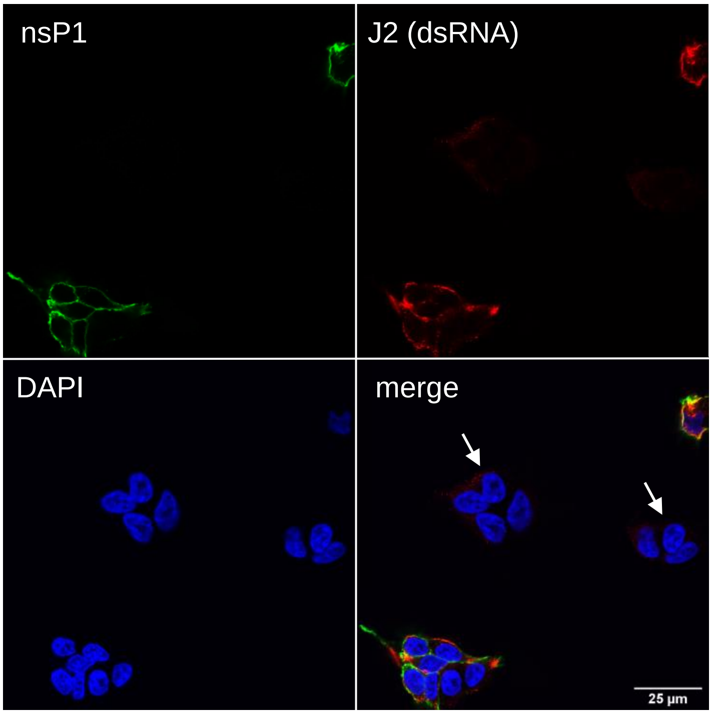

Supplementary Fig. 4. J2 antibody recognises viral RNA in CHIKV-infected HEK293T cells. Cells were infected for 18 hr at an MOI of 4. Infected cells were identified by labelling the CHIKV membrane-associated nonstructural protein 1 (nsP1). White arrows point to clusters of non-infected cells.

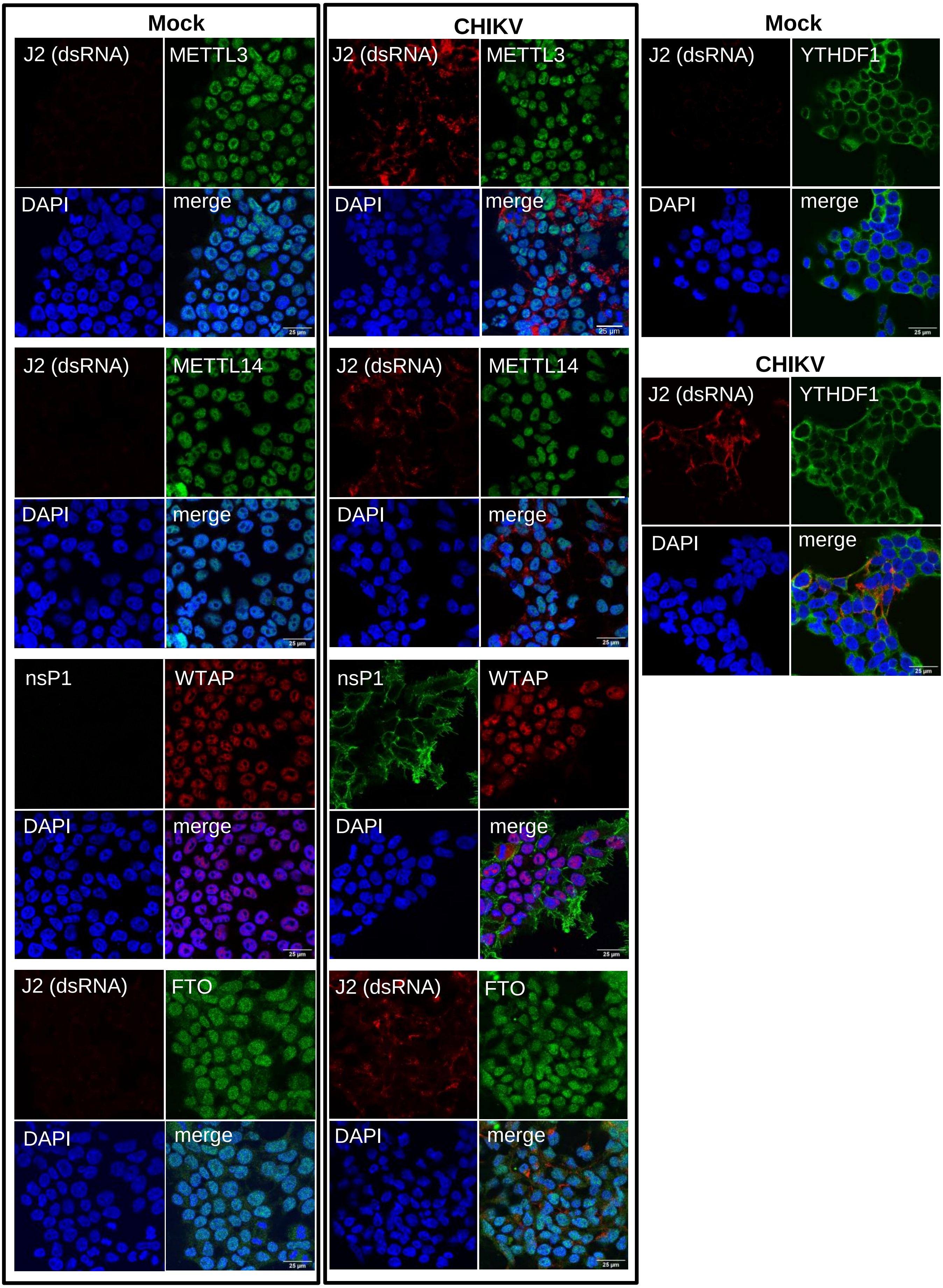

Supplementary Fig. 5. Localization of METTL3, METTL14, WTAP, FTO, or YTHDF1 in mock- or CHIKV-infected HEK293T cells. Cells were infected for 18 hr at an MOI of 4. Note that antibodies against METTL3, METTL14, FTO, YTHDF1, and nsP1 are derived from rabbits, while J2 and WTAP are derived from mice.

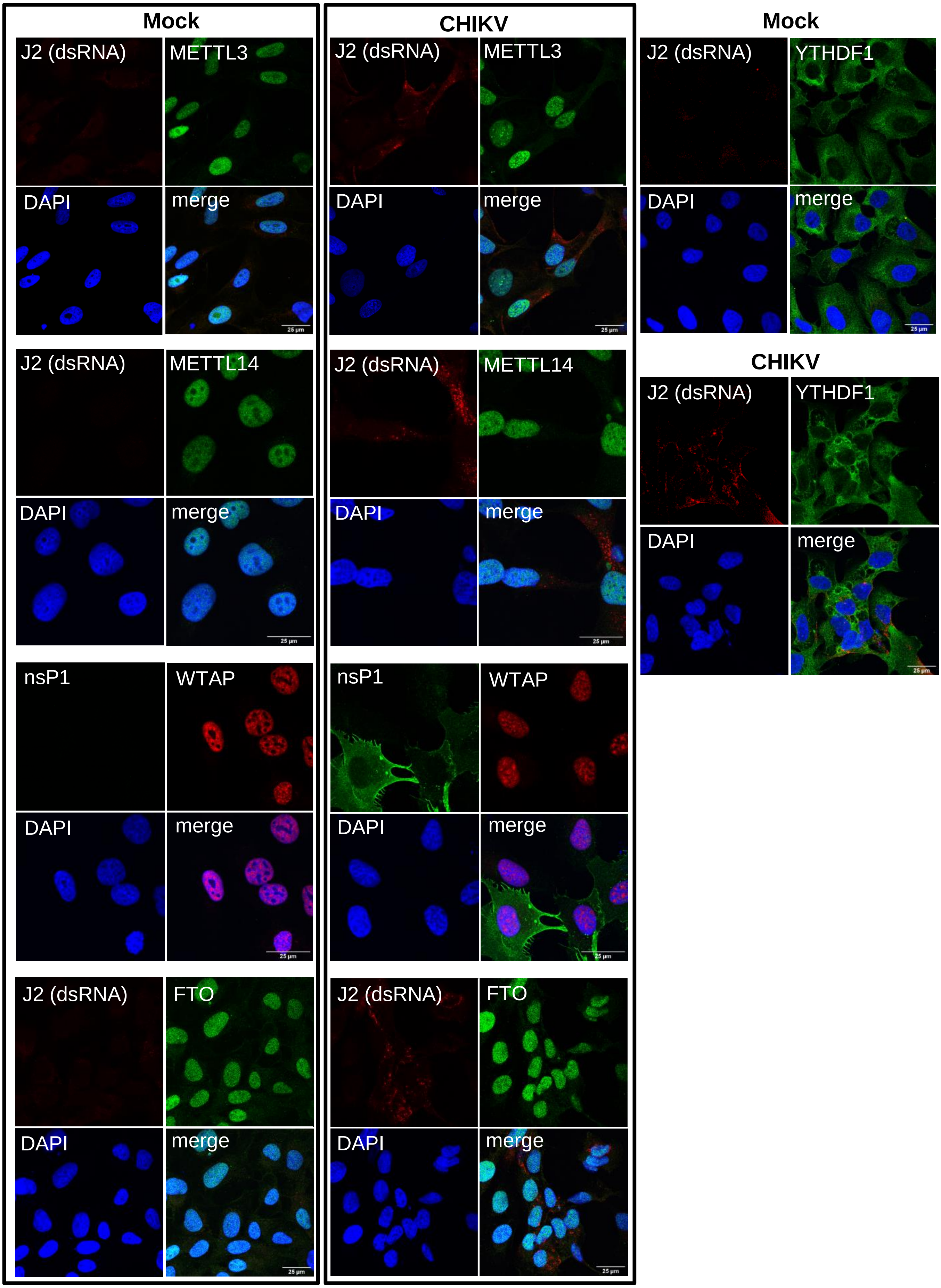

Supplementary Fig. 6. Localization of METTL3, METTL14, WTAP, FTO, or YTHDF1 in mock- or CHIKV-infected U2OS cells. Cells were infected for 18 hr at an MOI of 4. Note that antibodies against METTL3, METTL14, FTO, YTHDF1, and nsP1 are all derived from rabbits while J2 and WTAP are derived from mice.

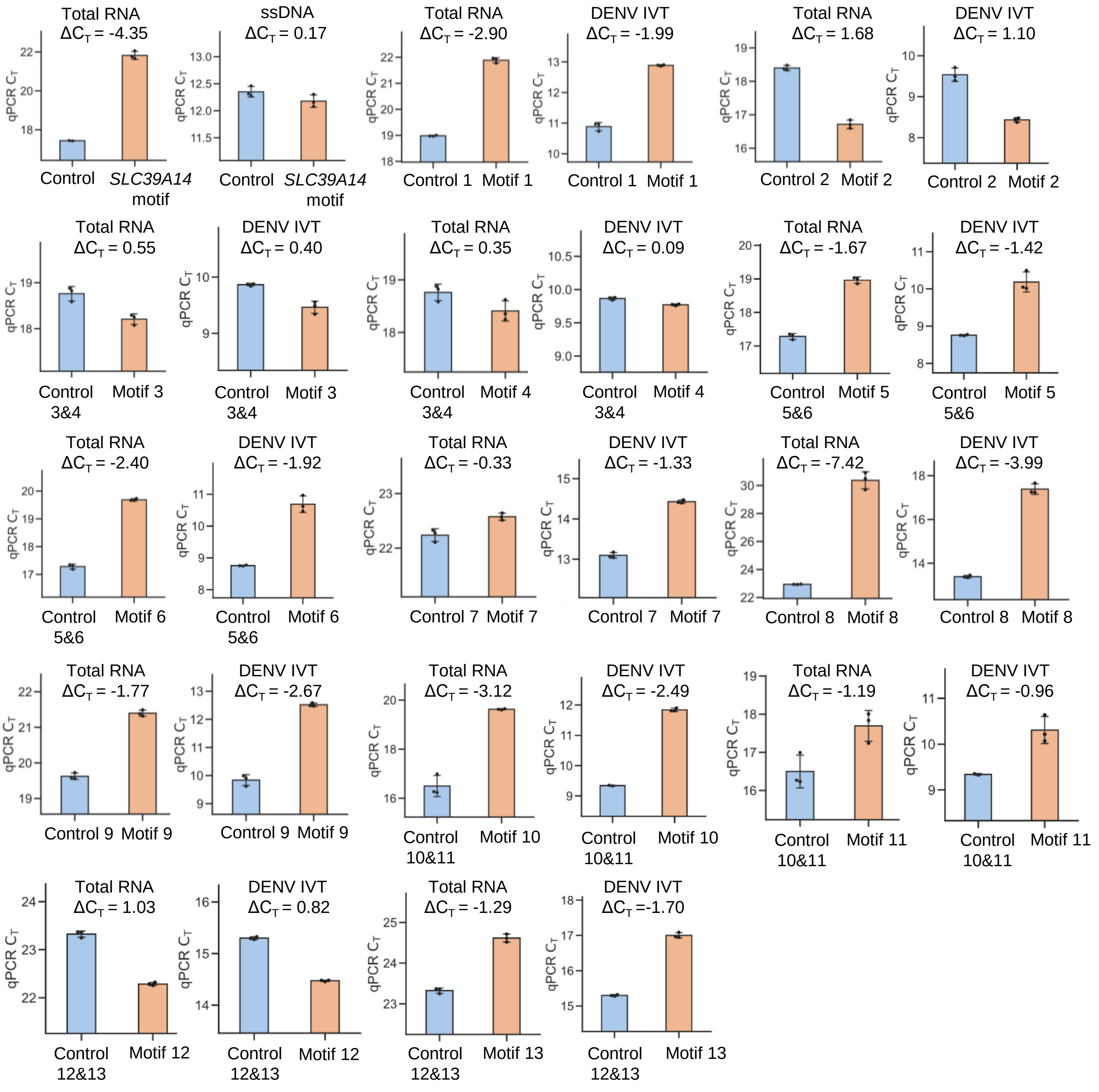

Supplementary Fig. 7. Raw C_T cycles from SELECT analyses of DENV RNAs in Huh7 cells. The bar chart shows mean values from three SELECT reactions with the error bars showing SD.

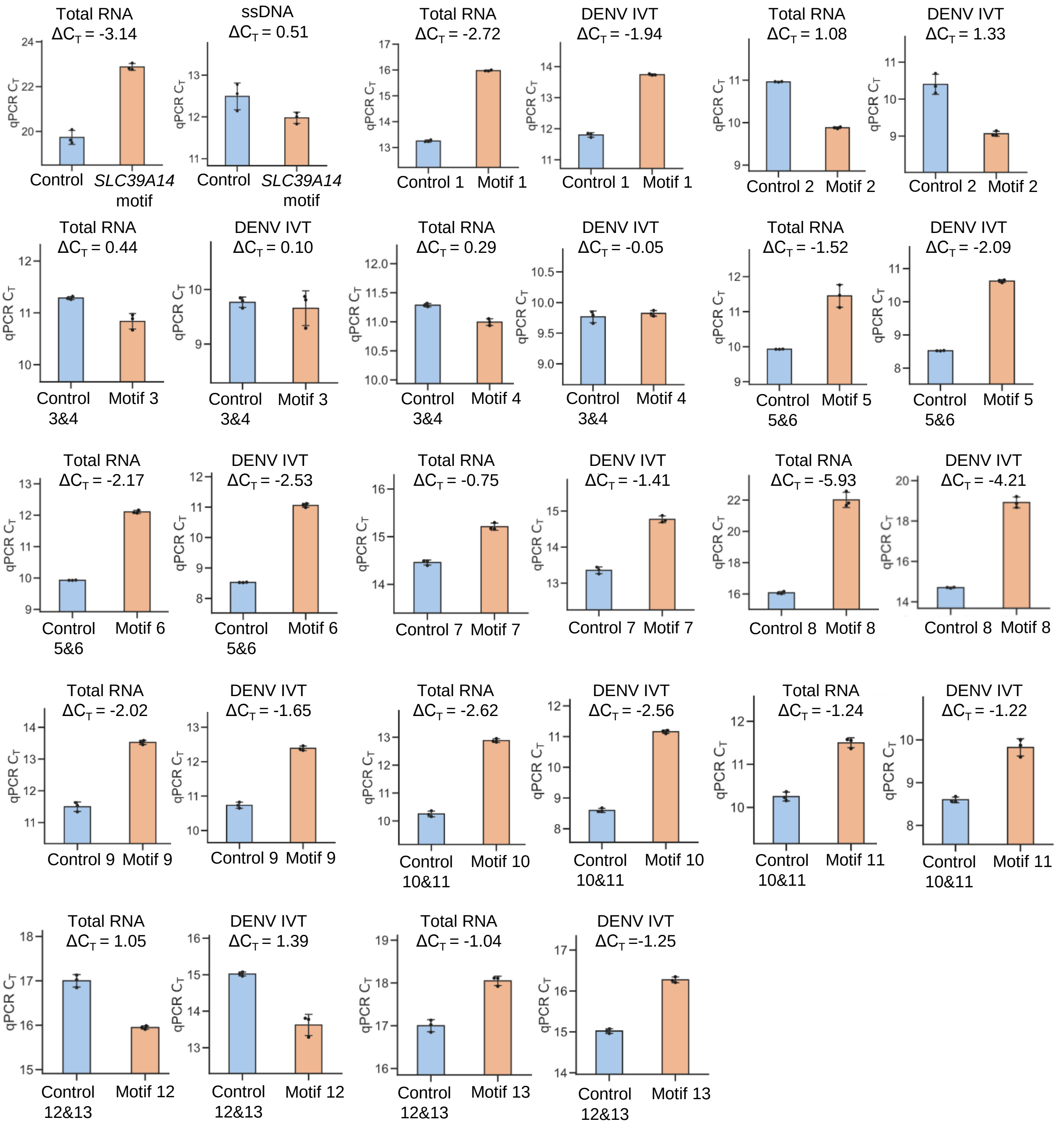

Supplementary Fig. 8. Raw C_T cycles from SELECT analyses of DENV RNAs in HEK293T cells. The bar chart shows mean values from three SELECT reactions with the error bars showing SD.

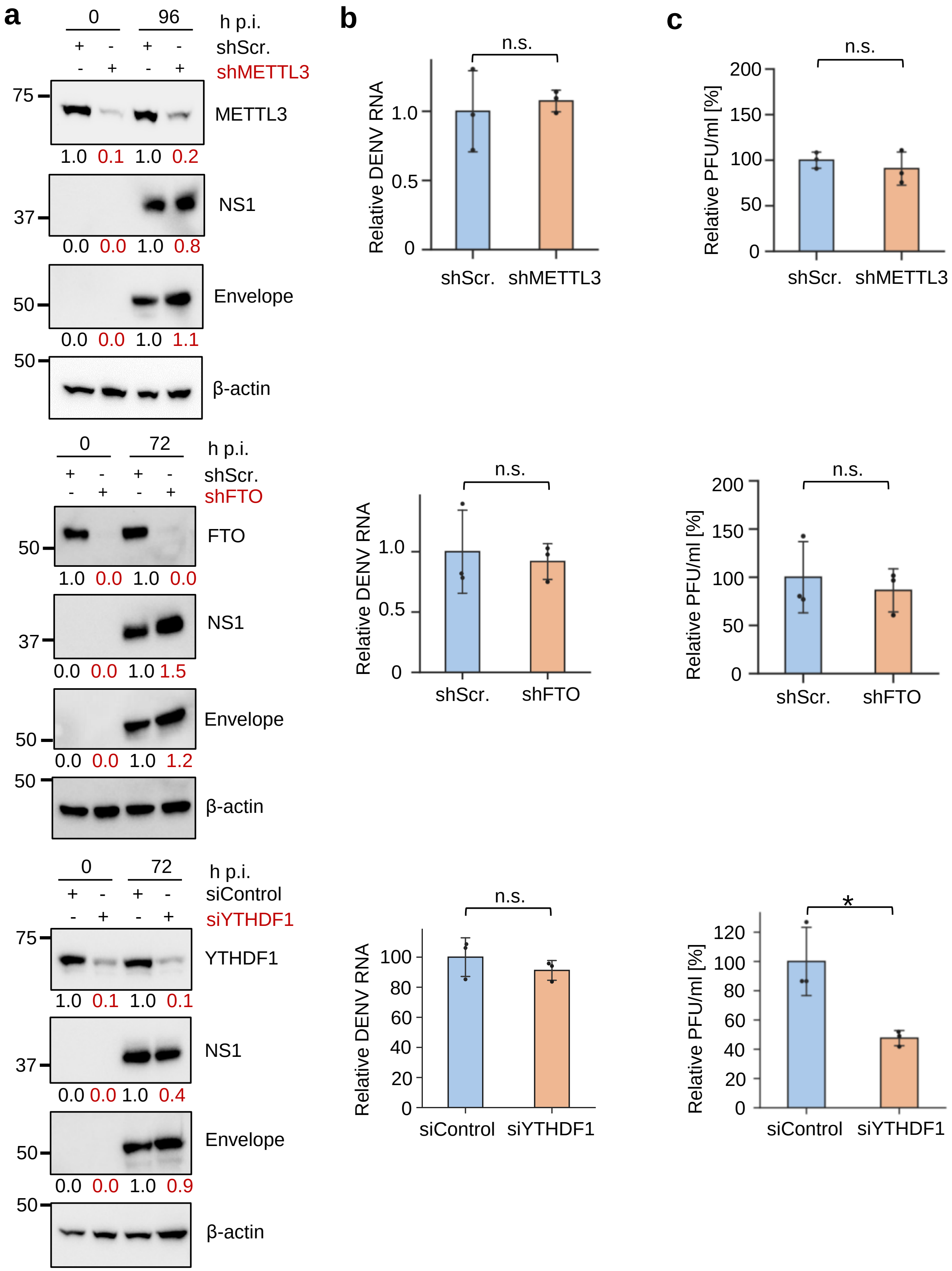

Supplementary Fig. 9. Effect of depletion of METTL3, FTO, or YTHDF1 on DENV infection. Stable shRNA HEK293T cell lines were infected at an MOI of 0.5 for the indicated time post-infection. At 24 hr of siRNA depletion, siRNA-treated HEK293T cells were infected for 72 hr at an MOI of 0.5. Scr. = scramble, h p.i. = hours post-infection. n.s. = not significant. **a)** Western blot quantification analyses are representative from 2 independent infections. β-actin-normalised values from depleted samples, below each band, are shown relative to their controls. Uncropped blots in Source Data. **b)** Intracellular viral RNA levels were quantified by qRT-PCR and normalised against the housekeeping gene *GAPDH*. DENV RNA levels in depleted samples are shown relative to their corresponding control. The bar chart shows mean values from 3 independent infections with the error bars showing SD. **c)** Supernatants collected 72 h p.i. from DENV-infected control and knockdown cells were titered by plaque assay in HEK293T cells. The bar chart shows relative mean values from 3 independent replicates with the error bars showing SD. All statistical analyses were performed using a two-tailed *t*-test. n.s. = not significant.

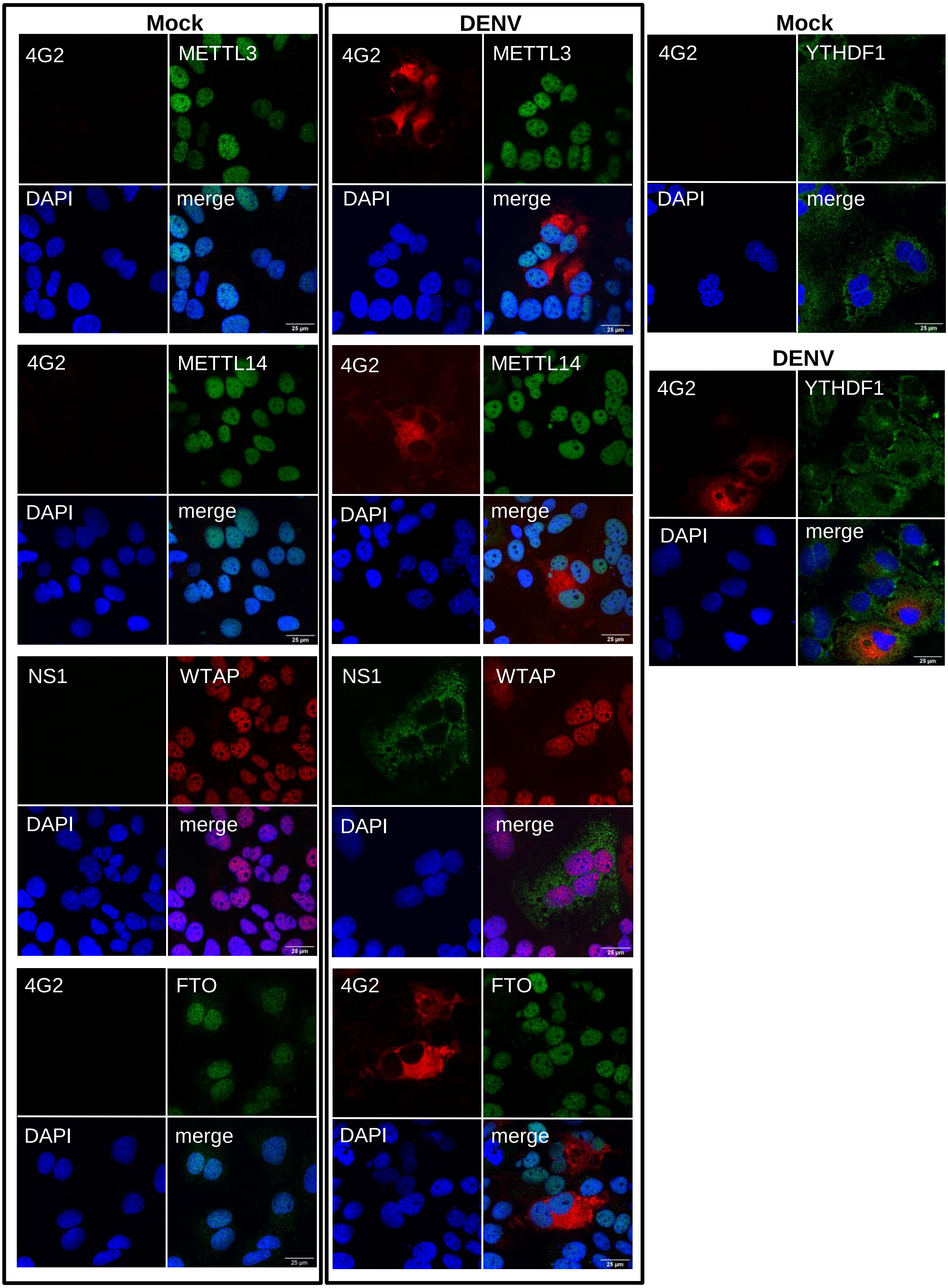

Supplementary Fig. 10. Localization of METTL3, METTL14, WTAP, FTO, or YTHDF1 in mock- or DENV-infected Huh7 cells. Cells were infected for 48 hr at an MOI of 3. 4G2 antibody recognises the DENV-2 envelope protein (E protein). NS1 antibody recognises the DENV-2 nonstructural protein 1 (NS1). Note that antibodies against METTL3, METTL14, FTO, YTHDF1, and NS1 are all derived from rabbits while WTAP and 4G2 are derived from mouse.

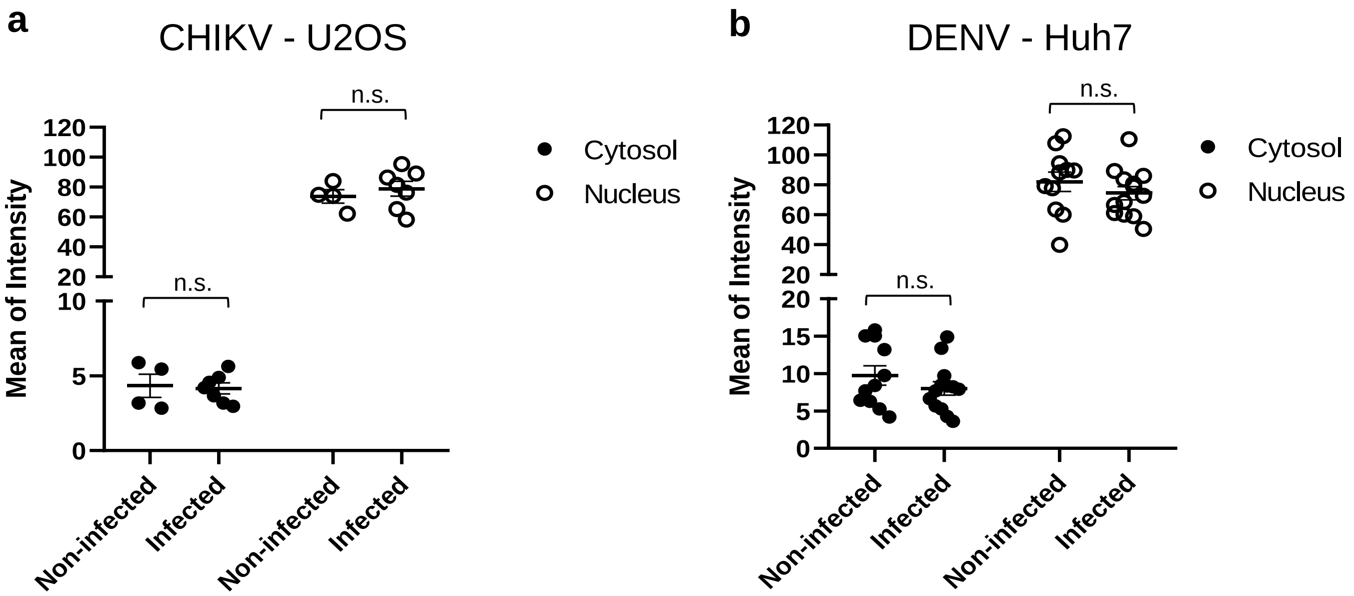

Supplementary Fig. 11. Quantification of the nuclear and cytoplasmic fluorescent signal for WTAP, in non-infected versus infected cells. a) Confocal micrographs of mock- or CHIKV (18 h p.i., MOI of 4)-infected U2OS cells were used for quantification. n = 4 for non-infected and 7 for infected. **b)** Confocal micrographs of mock- or DENV (48 h p.i., MOI of 3)-infected Huh7 cells were used for quantification. n = 11 for non-infected and 13 for infected. Graphs show the mean (of all mean gray values) with error bars representing standard error of the mean (SEM). All statistical analyses were performed using a two-tailed *t*-test. n.s. = not significant.

m6A-seq dataset	Sample	Raw reads	Uniquely mapped (total)	Uniquely mapped (Virus)	% Viral Reads
CHIKV polyA+	Input 1	18,056,909	14,672,482	6,138,459	42%
	Input 2	17,239,318	14,117,539	8,113,041	57%
	m6A-IP 1	17,694,296	15,101,890	2,517,543	17%
	m6A-IP 2	15,626,718	11,586,734	2,484,389	21%
Dengue Baquero	Input 1	71,557,021	50,323,684	3,425,804	7%
	Input 2	61,674,100	40,904,351	714,475	2%
	m6A-IP 1	65,258,138	56,593,150	1,752,938	3%
	m6A-IP 2	55,873,036	47,939,668	286,583	1%
Dengue Gokhale 2016	Input 1	136,803,826	109,712,125	1,683,461	2%
	Input 2	106,398,900	82,338,472	1,043,059	1%
	m6A-IP 1	162,044,085	127,031,644	2,299,743	2%
	m6A-IP 2	174,885,561	136,683,051	1,839,113	1%
Dengue Gokhale 2020	Input 1	58,097,265	37,225,481	4,186,036	11%
	Input 2	65,256,777	32,419,434	4,005,003	12%
	Input 3	78,565,261	45,799,265	3,718,021	8%
	m6A-IP 1	65,937,997	34,434,342	373,351	1%
	m6A-IP 2	55,081,342	44,262,807	924,741	2%
	m6A-IP 3	63,087,590	55,393,604	412,209	1%

m6A-seq dataset	Sample	Raw reads	Uniquely mapped (total)	Uniquely mapped to SLC39A14	% Host Reads
CHIKV polyA+	Input 1	18,056,909	14,672,482	679	0%
	Input 2	17,239,318	14,117,539	409	0%
	m6A-IP 1	17,694,296	15,101,890	2,240	0%
	m6A-IP 2	15,626,718	11,586,734	1,548	0%
Dengue Baquero	Input 1	71,557,021	50,323,684	16,061	0%
	Input 2	61,674,100	40,904,351	13,784	0%
	m6A-IP 1	65,258,138	56,593,150	46,832	0%
	m6A-IP 2	55,873,036	47,939,668	35,753	0%

Supplementary table 1. m^6A-Seq mapping statistics

runID	Sample ID	Virus	Cell type	Condition	Replicate	Barcode	Sequenced reads	Mapped reads to viral genome	Median length mapped reads to the viral genome (bp)	Assigned to genomic transcript [a]	Assigned to subgenomic transcript [b]	ENA
RNA010420191	CHK-IVT-Rep1	CHIKV	NA	IVT	rep1	NA	16648	1102	1454	NA	NA	PRJEB61652
RNA030323	CHK-IVT-Rep2	CHIKV	NA	IVT	rep2	NA	13704	5401	938.5	NA	NA	
RNA140420	CHK-HEK-Rep1	CHIKV	HEK293T	shSCR	rep1	NA	2127412	1050330	886	27719	46023	
RNA160620	CHK-HEK-Rep2	CHIKV	HEK293T	shSCR	rep2	BC2	300066	129896	1035	2548	5856	
RNA160620	CHK-HEK-Rep3	CHIKV	HEK293T	WT	rep3	BC3 + BC4	182920	137373	664	1270	1586	

[a] all reads whose length is equal or larger than 4517bp were considered genomic transcripts.

[b] all reads whose alignment coordinates start in 7567-8067 and end in position 11700 bp or larger were considered subgenomic transcripts.

Supplementary table 2. Nanopore DRS statistics

Type of sample	Sample 1	Sample 2	NanoConsensus with default parameters (--MZS_thr 5 --NC_thr 5)				NanoConsensus with non-stringent parameters (--MZS_thr 3.75 --NC_thr 4)			
			**Number of sites in the viral transcripts**	**Number of replicable sites**	**Replicable sites (%)**	**Replicable sites with DRACH motif**	**Number of sites in the viral transcripts**	**Number of replicable sites**	**Replicable sites (%)**	**Replicable sites with DRACH motif**
Full viral DRS datasets	CHK-IVT-Rep1	CHK-HEK-Rep1	1	0	0.00	0	12	2	8.70	1
	CHK-IVT-Rep2	CHK-HEK-Rep2	3				13			
	CHK-IVT-Rep1	CHK-IVT-Rep2	3	NA	NA	NA	31	NA	NA	NA
	CHK-HEK-Rep1	CHK-HEK-Rep2	12	NA	NA	NA	33	NA	NA	NA
Full-length genomic or subgenomic transcripts	CHK-IVT-Rep1	CHK-HEK2-Genomic	6	0	0.00	0	23	2	4.88	1
	CHK-IVT-Rep2	CHK-HEK3-Genomic	3				20			
	CHK-IVT-Rep1	CHK-HEK2-Subgenomic	10	0	0.00	0	8	1	7.14	1
	CHK-IVT-Rep2	CHK-HEK3-Subgenomic	3				7			
	CHK-HEK2-Genomic	CHK-HEK3-Genomic	4	NA	NA	NA	19	NA	NA	NA
	CHK-HEK2-Subgenomic	CHK-HEK3-Subgenomic	4	NA	NA	NA	7	NA	NA	NA

Paired-comparisons served as negative controls

Supplementary table 3. Summary of NanoConsensus results

runID	Sample ID	Virus	Cell type	Condition	Replicate	Barcode	Sequenced reads	Mapped reads to host genome	Mean length mapped reads to the host genome (bp)	ENA
NA	Host-IVT-Rep1	NA	HEK	IVT	rep1	NA	1338412	715133	941	PRJNA777450
NA	Host-IVT-Rep2	NA	HEK	IVT	rep2	NA	1801774	1117552	983	
RNA140420	CHK-HEK-Rep1	CHK	HEK	WT	rep1	NA	2127412	879691	825	PRJEB61652
RNA160620	CHK-HEK-Rep2	CHK	HEK	WT	rep2	BC2	300066	143691	816	

Supplementary table 4. Nanopore DRS host sequencing statistics

Ribonucleoside	[MH+]
C	244.09
U_Y	245.08
A	268.1
m1A_m2A_m6A_m8A_Am	282.12
G	284.1

List of all the modified ribonucleosides identified in the mass spectrometry analyses with its corresponding precursor [MH+].

Supplementary table 5. Table of modified ribonucleosides identified in mass spectrometry

Short name	Company	Reference
**C**	Carbosynth	NC04070
**U**	Carbosynth	NU06309
**A**	Carbosynth	NA01639
**m6A**	Carbosynth	NM32281
**G**	Carbosynth	NG06314

Supplementary table 6. Catalogue numbers of commercial ribonucleosides
