# Supplementary material for: N6-methyladenosine modification is not a general trait of viral RNA genomes
Source: Nat Commun. 2024 Mar 11;15:1964. doi: 10.1038/s41467-024-46278-9 (PMC10928186; doi:10.1038/s41467-024-46278-9)
Supplement: Supplementary file 3 — Description of Additional Supplementary Files [file 41467_2024_46278_MOESM3_ESM.pdf]

Description of Additional Supplementary Files

Supplementary Data 1

Tittle: List of primers and oligos

Description: Sequences of primers and oligos used in this study.

Supplementary Data 2

Title: List of called m6A peaks

Description: List of called cellular m6A peaks in our CHIKV and DENV datasets. List of viral m6A peaks called in our CHIKV and DENV datasets and Gokhale's DENV datasets.
