# Supplementary material for: N6-methyladenosine modification is not a general trait of viral RNA genomes
Source: Nat Commun. 2024 Mar 11;15:1964. doi: 10.1038/s41467-024-46278-9 (PMC10928186; doi:10.1038/s41467-024-46278-9)
Supplement: Supplementary file 6 — Reporting Summary [file 41467_2024_46278_MOESM6_ESM.pdf]

nature portfolio

Corresponding author(s): Juana Díez, Eva Maria Novoa and Stuard Wilson

Last updated by author(s): Feb 6, 2024

Reporting Summary

Nature Portfolio wishes to improve the reproducibility of the work that we publish. This form provides structure for consistency and transparency in reporting. For further information on Nature Portfolio policies, see our Editorial Policies and the Editorial Policy Checklist.

Statistics

For all statistical analyses, confirm that the following items are present in the figure legend, table legend, main text, or Methods section.

n/a	Confirmed	
☐	☒	The exact sample size (*n*) for each experimental group/condition, given as a discrete number and unit of measurement
☐	☒	A statement on whether measurements were taken from distinct samples or whether the same sample was measured repeatedly
☐	☒	The statistical test(s) used AND whether they are one- or two-sided<br>*Only common tests should be described solely by name; describe more complex techniques in the Methods section.*
☒	☐	A description of all covariates tested
☐	☒	A description of any assumptions or corrections, such as tests of normality and adjustment for multiple comparisons
☐	☒	A full description of the statistical parameters including central tendency (e.g. means) or other basic estimates (e.g. regression coefficient) AND variation (e.g. standard deviation) or associated estimates of uncertainty (e.g. confidence intervals)
☐	☒	For null hypothesis testing, the test statistic (e.g. *F*, *t*, *r*) with confidence intervals, effect sizes, degrees of freedom and *P* value noted<br>*Give P values as exact values whenever suitable.*
☒	☐	For Bayesian analysis, information on the choice of priors and Markov chain Monte Carlo settings
☒	☐	For hierarchical and complex designs, identification of the appropriate level for tests and full reporting of outcomes
☒	☐	Estimates of effect sizes (e.g. Cohen's *d*, Pearson's *r*), indicating how they were calculated

Our web collection on statistics for biologists contains articles on many of the points above.

Software and code

Policy information about availability of computer code

Data collection	No custom software was used.
Data analysis	For the m6A-seq analysis, the following softwares were used:<br>- Preprocessing: Cutadapt v1.9.1, parameters: –e 0.05 -q 20 –minimum-length 25<br>- Mapping: STAR v.2.7.6a, parameters: --outFilterType BySJout<br>- Read counting statistics: samtools v.1.9, parameters: view -F 3844| grep NH:i:1<br>- Isolation of viral reads for CPM normalization and peakcalling: samtools v.1.9, command: view -h | awk '{if($3 != "viral genome"){print $0}}' | samtools view -Shb - > outfile<br>- Peakcalling: macs2 v.2.2.7.1, parameters: -q 0.01 –nomodel –extsize 100 -B –SPMR –bdg –keep-dup all -f BAMPE (-f BAM for public SE data)<br>- Peakcalling: m6aViewer v.1.6.1, default parameters<br>- CPM normalized track generation: Deeptools v3.5.1, command: bamCoverage --binSize 1 --exactScaling --normalizeUsing CPM --effectiveGenomeSize 10723 (viral) / 2913022398 (human)<br>- LFC track generation: macs2 v.2.2.7.1, parameters: bdgcmp -m FE<br>- Motif enrichment: HOMER v.4.11, command: findMotifsGenome.pl -rna -len 5,6 -size 50<br>- Downstream statistical analyses: peaks replicate intersection, q < 0.01 ; p <0.05 filtering : R v.4.2.0<br><br>For the analysis of direct RNA sequencing data:<br>All code used to analyze the runs are publicly available on GitHub (https://github.com/novoalab/DRS_CHIKV_Analysis and https://github.com/novoalab/NanoConsensus).<br>- Direct RNA reads were basecalled using Guppy basecaller v3.1.5 in high-accuracy (hac) mode

- Basecalled reads were mapped using minimap2 v2.17 or graphmap v0.5.2.
- NanoConsensus code and documentation is publicly available in GitHub (https://github.com/novoalab/NanoConsensus) and in Zenodo (https://doi.org/10.5281/zenodo.5805806).
- All analysis workflow is publicly available in https://github.com/novoalab/DRS_CHIKV_Analysis (https://doi.org/10.5281/zenodo.10555493).

To analyse gel images, these softwares below were used:
- Image gauge software FIJI_ImageJ win64 v1.54f
- Image Lab (BIO-RAD) version 6.0.1 build 7

For manuscripts utilizing custom algorithms or software that are central to the research but not yet described in published literature, software must be made available to editors and reviewers. We strongly encourage code deposition in a community repository (e.g. GitHub). See the Nature Portfolio guidelines for submitting code & software for further information.

Data

Policy information about availability of data

All manuscripts must include a data availability statement. This statement should provide the following information, where applicable:

- Accession codes, unique identifiers, or web links for publicly available datasets
- A description of any restrictions on data availability
- For clinical datasets or third party data, please ensure that the statement adheres to our policy

Data supporting the findings of this study are available within the article and its Supplementary Figures and Tables. Source data is provided as a Source Data files. The raw mass spectrometry data have been deposited to the MetaboLights repository with the dataset MTBLS6978 (www.ebi.ac.uk/metabolights/MTBLS6978).

The m6A-seq datasets from this study have been submitted to the NCBI Gene Expression Omnibus (GEO; http://www.ncbi.nlm.nih.gov/geo/) under accession number GSE231739. Base-called fast5 nanopore direct RNA sequencing data have been deposited at ENA under the accession PRJEB61652 (https://www.ebi.ac.uk/ena/browser/view/PRJEB61652).

Publicly available datasets analyzed as part of this study:
- M6A-seq in DENV infected Huh7 cells (GEO): GSM2203041(https://www.ncbi.nlm.nih.gov/geo/query/acc.cgi?acc=GSM2203041), GSM2203042 (https://www.ncbi.nlm.nih.gov/geo/query/acc.cgi?acc=GSM2203042), GSM2203043 (https://www.ncbi.nlm.nih.gov/geo/query/acc.cgi?acc=GSM2203043), GSM2203044 (https://www.ncbi.nlm.nih.gov/geo/query/acc.cgi?acc=GSM2203044).
- M6A-seq in DENV infected Huh7 cells (GEO): GSM3755800 (https://www.ncbi.nlm.nih.gov/geo/query/acc.cgi?acc=GSM3755800), GSM3755801 (https://www.ncbi.nlm.nih.gov/geo/query/acc.cgi?acc=GSM3755801), GSM3755802 (https://www.ncbi.nlm.nih.gov/geo/query/acc.cgi?acc=GSM3755802), GSM3755803 (https://www.ncbi.nlm.nih.gov/geo/query/acc.cgi?acc=GSM3755803), GSM3755805 (https://www.ncbi.nlm.nih.gov/geo/query/acc.cgi?acc=GSM3755805), GSM3755806 (https://www.ncbi.nlm.nih.gov/geo/query/acc.cgi?acc=GSM3755806).
- PA-m6A-seq (GEO): GSM1326564 (https://www.ncbi.nlm.nih.gov/geo/query/acc.cgi?acc=GSM1326564), GSM1326565 (https://www.ncbi.nlm.nih.gov/geo/query/acc.cgi?acc=GSM1326565).
- Nanopore direct RNA sequencing runs in A549 wild type and METTL3 KO Ad5-infected cells (ENA): PRJEB35652 (https://www.ebi.ac.uk/ena/browser/view/PRJEB61652).
- NGS-based plasmid DNA sequencing data from this study have been deposited at ENA under the accession PRJEB66350 [https://www.ebi.ac.uk/ena/browser/view/PRJEB66350].

References used for the analysis are the ones below:
- Human hg38 genome FASTA (https://hgdownload.soe.ucsc.edu/goldenPath/hg38/bigZips/hg38.fa.gz)
- CHIKV genome FASTA & annotation (https://www.ncbi.nlm.nih.gov/nuccore/DQ443544)
- DENV genome FASTA & annotation (https://www.ncbi.nlm.nih.gov/nuccore/158976983)

Research involving human participants, their data, or biological material

Policy information about studies with human participants or human data. See also policy information about sex, gender (identity/presentation), and sexual orientation and race, ethnicity and racism.

Reporting on sex and gender	Our study exclusively made use of cell lines and none of these were established from primary cells of humans or vertebrate models. Source and gender of the cell lines is reported in the section 'Eukaryotic cell lines'.
Reporting on race, ethnicity, or other socially relevant groupings	NA
Population characteristics	NA
Recruitment	NA
Ethics oversight	NA

Note that full information on the approval of the study protocol must also be provided in the manuscript.

Field-specific reporting

Life sciences study design

All studies must disclose on these points even when the disclosure is negative.

Sample size	In general, no calculations were done to determine sample size. Sample size was determined based on standards for experimental studies attempting to have a minimum of n=3 biological replicates with sufficient reproducibility.
Data exclusions	No data exclusions were performed.
Replication	The results were confirmed by performing the experiment multiple times with successful replication (at least in three independent replicates) in conjunction with complementary techniques.
Randomization	Samples were allocated randomly for culture, infection, and analysis.
Blinding	Blinding was not performed in our study. The experimental parameters determined in this study are considered as objective measures, no subjected to bias and therefore the integrity of the results are not impacted when running the study and analysis unblinded.

Reporting for specific materials, systems and methods

We require information from authors about some types of materials, experimental systems and methods used in many studies. Here, indicate whether each material, system or method listed is relevant to your study. If you are not sure if a list item applies to your research, read the appropriate section before selecting a response.

Materials & experimental systems

n/a	Involved in the study	
☐	☒	Antibodies
☐	☒	Eukaryotic cell lines
☒	☐	Palaeontology and archaeology
☒	☐	Animals and other organisms
☒	☐	Clinical data
☒	☐	Dual use research of concern
☒	☐	Plants

Methods

n/a	Involved in the study	
☒	☐	ChIP-seq
☒	☐	Flow cytometry
☒	☐	MRI-based neuroimaging

Antibodies

Antibodies used

- Rabbit polyclonal anti-m6A antibody (Active motif, 61495). Lot number: 35720002.
- Anti-METTL3 (Abcam, ab195352) rabbit monoclonal [EPR18810]. Lot number: GR3375496-10.
- Anti-FTO (Abcam, ab126605) rabbit monoclonal [EPR6894]. Lot number: GR3180347-5.
- Anti-YTHDF1 polyclonal antibody (Proteintech, 17479–1-AP). Lot number: 00082562.
- Anti-β-actin (Sigma-Aldrich, A5441-.2ML). Clone AC-15. We were unable to trace the lot number.
- Anti-CHIKV capsid rabbit polyclonal antibody: kindly provided (not commercially available) by Prof. A. Merits (University of Tartu).
- Anti-flavivirus group antigen (Novus Biologicals, NBP2-52709-0.2mg) Clone: D1-4G2-4-15 (4G2). Lot number: T1911A06.
- Anti-DENV NS1 polyclonal antibody (GeneTex, GTX124280). Lot number: 41808.
- Anti-METTL14 polyclonal antibody (Sigma-Aldrich, HPA038002). Lot number: 000007978.
- Anti-WTAP (Proteintech, 60188-1-Ig) clone: 4A10G9. Lot number: 10004947.
- Anti-double-stranded RNA (Clone J2) (Nordic-MUbio, 10010200). Lot number: J2-2102.
- Horseradish peroxidase-conjugated secondary antibodies: anti-mouse (NA931-1ML, lot number: 17621142) and anti-rabbit (NA934-1ML, lot number: 17434242), both from Sigma-Aldrich.
- Fluorescently conjugated secondary antibodies: Alexa Fluor 488 goat anti-rabbit IgG (Thermo Fisher, A-11008, lot number: 2382186) and Alexa Fluor 568 goat anti-mouse IgG (Thermo Fisher, A-11004, lot number: 2332536).

Validation

- Rabbit anti-m6A antibody (Active motif, 61495) was validated in this study for use in m6A-RNA immunoprecipitation, including m6A-IP-qRT-PCR and m6A-Seq. Applications validated by Active Motif: dot blot. This antibody has also been published using dot blot (https://pubmed.ncbi.nlm.nih.gov/30982744/).

- Anti-METTL3 antibody [EPR18810] (Abcam, ab195352) has been validated by Abcam in IP, WB, IF, Flow cytometry and IHC-P and tested in human samples. In this study has been validated for use in WB and IF and multiple articles use this antibody for the same applications (https://www.abcam.com/en-es/products/primary-antibodies/mettl3-antibody-epr18810-ab195352#drawer=publications).

- Anti-FTO antibody [EPR6894] (Abcam, ab126605) has been validated by Abcam in WB, IF and IHC-P and tested in human samples. In this study has been validated for use in WB and IF. Multiple articles use this antibody for the same applications (https://www.abcam.com/en-es/products/primary-antibodies/fto-antibody-epr6894-ab126605#drawer=publications). FTO antibody was also validated via KD and detection of endogenous protein levels by WB in previous studies (Baquero-Pérez et al. eLife 8, e47261, 2019;

https://doi.org/10.7554/eLife.47261).

- Anti-YTHDF1 antibody (Proteintech, 17479-1-AP) has been validated by Proteintech in WB, RIP, IP, IHC, IF, FC, CoIP, ELISA and tested in human samples. In this study has been validated for use in WB and IF. YTHDF1 antibody was also validated via KD and detection of endogenous protein levels by WB in previous studies (Baquero-Pérez et al. eLife 8, e47261, 2019; https://doi.org/10.7554/eLife.47261). Multiple studies also validate this antibody for use in WB and IF (https://www.ptglab.com/products/YTHDF1-Antibody-17479-1-AP.html).

- Anti-β-actin antibody (Sigma-Aldrich, A5441-.2ML) was validated by Sigma-Aldrich for use in WB and IF using human samples. Relevant references from the provider: North, A.J., et al., J. Cell Biol., 120, 1159-67 (1993). Drew, J., et al., Amer. J. Physiol., 260, C1332 (1991).

- Anti-CHIKV capsid was validated in previous studies for IF and WB (Taylor et al, ASM Journals, 2017, Vol.8, No.1; https://doi.org/10.1128/mBio.01970-16) (Jungfleisch, J. et al. Nature Communications 13, 4725, 2022. https://doi.org/10.1038/s41467-022-31835-x). This antibody was also validated in this study for use in WB and IF.

- Anti-flavivirus group antigen (Novus Biologicals, NBP2-52709-0.2mg) is validated by Novus Biologicals for the following applications: WB, ELISA, Flow, ICC/IF, IHC, B/N, CyTOF-ready, ICC/IF. This antibody was also validated in previous studies for WB (Wollner et al. J Virol 95, e02482-02420, 2021; https://doi.org/10.1128/JVI.02482-20) and IF (Vogt et al. PLOS Neglected Tropical Diseases 13, e0007837, 2019; https://doi.org/10.1371/journal.pntd.0007837). In this study this antibody was also validated for use in WB and IF.

- Anti-DENV NS1 (GeneTex, GTX124280) was validated by GeneTex for use in WB, ICC/IF, IHC-P, FACS, IP, ELISA, IHC-P (cell pellet). Additionally, this antibody has been cited in the literature multiple times for use in WB (https://www.genetex.com/Product/Detail/Dengue-virus-NS1-protein-antibody/GTX124280#references). In this study it was validated for use in WB and IF.

- Anti-METTL14 (Sigma-Aldrich, HPA038002) was validated in previous studies for WB (Lin et al. Molecular Cell 62, 335-345, 2016; https://doi.org/10.1016/j.molcel.2016.03.021). This antibody was validated for use in IF by the Human Protein Atlas (HPA) project. This antibody is validated by the vendor to work in human samples in IF and IHC. In this study this antibody was validated for use in IF. Multiple articles have used this antibody for WB (https://www.sigmaaldrich.com/ES/es/product/sigma/hpa038002).

- Anti-WTAP (Proteintech, 60188-1-Ig) was validated by Proteintech for use in WB, IHC and IF. This antibody has been cited in many articles for use in WB https://www.ptglab.com/products/WTAP-Antibody-60188-1-Ig.htm#publications In this study, this antibody was validated for IF.

- Anti-double-stranded RNA (J2) (Nordic-MUbio, 10010200) was validated in previous studies for use in IF (Weber et al. J Virol 80, 5059-5064, 2006; https://doi.org/10.1128/JVI.80.10.5059-5064.2006). The antibody is validated by the vendor for Dot blot, dsRNA-immunoblotting, ELISA, Flow Cytometry, Immuno-affinity-chromatography, Immunocytochemistry and Immunohistochemistry. This antibody was validated in this study for use in IF.

Eukaryotic cell lines

Policy information about cell lines and Sex and Gender in Research

Cell line source(s)	- Human embryonic kidney cells (HEK293T). Obtained from ATCC (CRL-11268). Female.<br>- Huh7 are a gift from Prof. Francis Chisari, The Scripps Research Institute. Originally obtained from ThermoFisherScientific. Male.<br>- U2OS are a gift from Prof. Wolfram Brune, Leibniz Institute of Virology. Female.<br>- BHK-21 Clone 13. Obtained from ATCC (CCL-10). Male.
Authentication	Cell lines were not authenticated for this study.
Mycoplasma contamination	All cell lines were tested negative for mycoplasma contamination.
Commonly misidentified lines (See ICLAC register)	No commonly misindentified cell lines were used.

Plants

Seed stocks	*Report on the source of all seed stocks or other plant material used. If applicable, state the seed stock centre and catalogue number. If plant specimens were collected from the field, describe the collection location, date and sampling procedures.*
Novel plant genotypes	*Describe the methods by which all novel plant genotypes were produced. This includes those generated by transgenic approaches, gene editing, chemical/radiation-based mutagenesis and hybridization. For transgenic lines, describe the transformation method, the number of independent lines analyzed and the generation upon which experiments were performed. For gene-edited lines, describe the editor used, the endogenous sequence targeted for editing, the targeting guide RNA sequence (if applicable) and how the editor was applied.*
Authentication	*Describe any authentication procedures for each seed stock used or novel genotype generated. Describe any experiments used to assess the effect of a mutation and, where applicable, how potential secondary effects (e.g. second site T-DNA insertions, mosiacism, off-target gene editing) were examined.*
